# First detection of *Klebsiella variicola* producing OXA-181 carbapenemase in fresh vegetable imported from Asia to Switzerland

**DOI:** 10.1186/s13756-015-0080-5

**Published:** 2015-10-06

**Authors:** K. Zurfluh, L. Poirel, P. Nordmann, J. Klumpp, R. Stephan

**Affiliations:** Institute for Food Safety and Hygiene, Vetsuisse Faculty University of Zurich, Winterthurerstrasse 272, 8057 Zurich, Switzerland; Department of Medicine, Faculty of Science, Medical and Molecular Microbiology Unit “Emerging Antibiotic Resistance”, University of Fribourg, Fribourg, Switzerland; Institute of Food, Nutrition and Health, ETH Zürich, Schmelzbergstrasse 7, 8092 Zürich, Switzerland

**Keywords:** Carbapenemase, Food, Enterobacteriaceae, IncX3 plasmid, OXA-181

## Abstract

**Background:**

The emergence and worldwide spread of carbapenemase-producing Enterobacteriaceae is of great concern to public health services. The aim of this study was to investigate the occurrence of carbapenemase-producing Enterobacteriaceae in fresh vegetables and spices imported from Asia to Switzerland.

**Findings:**

Twenty-two different fresh vegetable samples were purchased in March 2015 from different retail shops specializing in Asian food. The vegetables included basil leaves, bergamont leaves, coriander, curry leaves, eggplant and okra (marrow). Samples had been imported from Thailand, the Socialist Republic of Vietnam and India. After an initial enrichment-step, carbapenemase-producing Enterobacteriaceae were isolated from two carbapenem-containing selective media (SUPERCARBA II and Brilliance CRE Agar). Isolates were screened by PCR for the presence of *bla*_KPC_, *bla*_NDM_, *bla*_OXA-48-like_ and *bla*_VIM_. An OXA-181-producing *Klebsiella variicola* was isolated in a coriander sample with origin Thailand/Vietnam. The *bla*_OXA-181_ gene was encoded in a 14′027 bp region flanked by two IS*26*-like elements on a 51-kb IncX3-type plasmid.

**Conclusions:**

The results of this study suggest that the international production and trade of fresh vegetables constitute a possible route for the spread of carbapenemase-producing Enterobacteriaceae. The presence of carbapenemase-producing organisms in the food supply is alarming and an important food safety issue.

## Findings

Carbapenemase-producing Enterobacteriaceae were first described in Europe in the 1990s, and since then they have been increasingly reported especially in clinical settings. Recently, the occurrence of *Escherichia coli* producing OXA-48 β-lactamase was also found for the first time in the community in Switzerland [1].

Currently, the most prevalent carbapenemases among Enterobacteriaceae include the Ambler class A carbapenemase KPC, the class B metallo-β-lactamases (MBLs) of the IMP-, NDM- or VIM-type, and the class D OXA-48-like oxacillinases [2].

The emergence and worldwide spread of carbapenemase-producing Enterobacteriaceae is of great concern to public health services. Here we describe for the first time the detection of carbapenemase-producing Enterobacteriaceae in a fresh produce ready-to-eat food sample.

For this study, 22 different fresh vegetable samples were purchased in March 2015 from ten different retail shops specializing in Asian food in three different cities in Switzerland. In eight shops each two samples and in two shops each three samples were purchased, respectively. The vegetables included basil leaves, bergamont leaves, coriander, curry leaves, eggplant, and okra (marrow). The selection of vegetable and spices were based on data of a previous study focusing on ESBL-producers [3]. Samples had been imported from Thailand (17 samples), the Socialist Republic of Vietnam (4 samples) and India (1 sample). One coriander sample was labeled as a mixed-sample with origin Thailand/Vietnam.

The fresh products were cooled right after purchase and immediately processed after arriving in the lab on the same day. Of each vegetable sample, 10–15 g were placed in a sterile Stomacher bag. Samples were homogenized using a Stomacher sample blender and incubated at ratio 1:10 in Enterobacteriaceae Enrichment (EE) broth (BD, Franklin Lakes, USA) at 37 °C over night. For the detection of carbapenemase-producing Enterobacteriaceae, SUPERCARBA II medium [1] selecting for bacteria with reduced susceptibility to ertapenem and Brilliance CRE agar (Oxoid, Hampshire, UK) were inoculated with one loopful of each of the enrichment cultures. Plates were incubated at 37 °C for 24 h under aerobic conditions.

Each plate with grown colonies was screened by PCR for the presence of *bla*_KPC_, *bla*_NDM_, *bla*_OXA-48-like_ and *bla*_VIM_ using primers described previously [4, 5]. From PCR positive plates single colonies were purified and retested by PCR. Species identification was performed using the API ID 32 E kit (bioMérieux, Geneva, Switzerland) and *rpoB* sequence analysis.

From a coriander mix sample imported from Thailand/Vietnam, a *Klebsiella variicola* strain (KS22), harbouring the *bla*_OXA-181_ carbapenemase gene (100 % identity after sequencing) was isolated. In order to evaluate whether the *bla*_OXA-181_ gene could be transferred, conjugation experiments were performed using a standardized method, as described elsewhere [6]. Conjugation of the *bla*_OXA-181_-carrying plasmid was not successful. Therefore, the plasmid was extracted using the Qiagen large construct extraction kit (Qiagen, Courtaboeuf, France) and transformated into *E. coli* DH5α by electroporation. Using a Pacific Biosciences SMRT approach, the whole sequence of plasmid pKS22-OXA-181 harbouring the *bla*_OXA-181_ gene was determined (accession number KT005457). The *bla*_OXA-181_ gene was encoded in a 14′027 bp region flanked by two IS*26*-like elements on an IncX3-type plasmid of 51′480 bp in size. The region also co-harboured a *qnrS1 gene.* The two IS*26*-like elements were in direct orientation and no duplicated target sequences have been identified on both extremities of the IS*26*-*bla*_OXA-181_-IS*26* fragment. Therefore, this fragment has been likely acquired through homologous recombination. Moreover, He et al. [7] recently suggested that IS*26* elements are also responsible for a large fraction of plasmid reorganizations by replicative transposition.

Susceptibility testing was performed for the *Klebsiella variicola* strain by the disk diffusion method (Becton Dickinson and Company, Maryland, USA) according to the manufacturers’ protocols. Minimal inhibitory concentrations (MIC) of imipenem, meropenem and ertapenem were determined by Etest strips (bioMérieux, Marcy l’Etoile, France). Results were interpreted according to the criteria of the Clinical and Laboratory Standards Institute (CLSI) [8].

For the *Klebsiella variicola* strain, the MIC’s of imipenem, meropenem and ertapenem were 0.38 μg/ml, 0.047 μg/ml and 0.064 μg/ml, which are below the resistance breakpoints defined by CLSI. Class D carbapenem-hydrolyzing β-lactamases possess weak carbapenemase-activity and usually do not confer a high-level resistance phenotype unless the strain exhibits additional permeability defects [9, 10]. Using the SUPERCARBA II medium, however, it was possible to detect a carbapenemase producer that is still considered sensitive according to the CLSI guidelines. This makes this selective medium appropriate for tracing carbapenemase producers regardless of any resistance, by contrast to some other selective media.

The *Klebsiella variicola* strain tested resistant in the disk diffusion test to ampicillin, amoxicllin-clavulanic acid, tetracycline and streptomycin. It was susceptible in the disk diffusion test to cefotaxime, sulfamethoxazole, trimethoprim, nalidixic acid, ciprofloxacin, gentamicin, chloramphenicol and kanamycin.

OXA-181, an OXA-48 type carbapenemase conferring resistance to penicillins and carbapenems, was first described 2007 in *E. cloacae* and *K. pneumoniae* strains isolated at several locations in India [11]. The gene *bla*_OXA-181_ originates from *Shewanella xiamenensis*, an environmental bacterium [12]. It is suggested that the main reservoir of *bla*_OXA-181_-harbouring Enterobacteriaceae is in the Indian subcontinent. Outside this subcontinent, Enterobacteriaceae isolates producing OXA-181 have sporadically been found in patients from several countries. However, for these patients normally a travelling history to the Indian subcontinent was reported.

So far the *bla*_OXA-181_ gene has been detected on a 7.6-kb ColE2-type plasmid [12] or on a 84-kb IncT plasmid [13]. Very recently, an OXA-181-producing *Escherichia coli* strain, harbouring the *bla*_OXA-181_ also on an IncX3 plasmid, was described in China [14]. The IncX3 plasmid pKS22-OXA-181 sequenced in this study and the plasmid pOXA181_EC14828, described for the *Escherichia coli* strain were nearly identical (with only two nucleotide differences in each of the IS*26* elements, position 316 C- > A, of the pKS22-OXA-181). The organization of the 14′027 bp region flanked by two IS*26*-like elements and harbouring the *bla*_OXA-181_ and an *qnrS* gene, was identical on both plasmids. Considering these similarities, it is highly probable that *bla*_OXA-181_ has started to spread on IncX3 plasmids.

In conclusion, the results of this study suggest that the international production and trade of fresh vegetables constitute a possible route for the spread of carbapenemase-producing Enterobacteriaceae, comparable to the situation recently found with extended spectrum β-lactamase-producing Enterobacteriaceae [3, 15]. The presence of carbapenemase-producing organisms in the food supply and in ready-to-eat food is alarming and constitutes a food safety issue. Appropriate measures such as the improvement of agricultural practices and water quality need to be taken and globally mandatory guidelines should be established in order to ensure consumer and public health worldwide. It is now even more important that further studies are carried out in order to survey the dissemination of carbapenemase-producing enterobacterial strains in the environment and the food chain.
